# Resveratrol alleviates obesity-induced skeletal muscle inflammation via decreasing M1 macrophage polarization and increasing the regulatory T cell population

**DOI:** 10.1038/s41598-020-60185-1

**Published:** 2020-03-02

**Authors:** Maryam Shabani, Asie Sadeghi, Hossein Hosseini, Maryam Teimouri, Reyhaneh Babaei Khorzoughi, Parvin Pasalar, Reza Meshkani

**Affiliations:** 1 Department of Clinical Biochemistry, Faculty of Medicine, Tehran University of Medical Sciences, Tehran, I.R. Iran; 2 Department of Clinical Biochemistry, Afzalipour School of Medicine, Kerman University of Medical Sciences, Kerman, Iran

**Keywords:** Chemokines, Cell polarity, Insulin signalling, Mechanisms of disease, Metabolic disorders

## Abstract

Resveratrol was reported to inhibit inflammatory responses; however, the role of this polyphenol in obesity-induced skeletal muscle inflammation remains unknown. Mice fed a high fat diet (HFD) were treated with resveratrol for 16 weeks. Resveratrol treatment decreased macrophage infiltration into skeletal muscle of HFD-fed mice. Resveratrol also led to the polarization of macrophages to the M2 direction, as well as decreasing the expression of a number of M1 pro-inflammatory cytokines [tumor necrosis factor α (TNF-α), interleukin 1 β (IL-1β) and interleukin 6 (IL-6)]. In addition, increased infiltration of regulatory T cells (Treg cells) was found following resveratrol treatment in skeletal muscle of mice. Decreased intramyocellular lipid deposition was associated with reduced expression levels of toll-like receptors 2 (TLR2) and TLR4 in resveratrol treated mice. We also found that diminished inflammation in skeletal muscle following resveratrol treatment was accompanied by increasing phosphorylation of 5’-adenosine monophosphate-activated protein kinase (AMPK) and decreasing phosphorylation of p38 mitogen-activated protein kinase (MAPK) and c-Jun N-terminal kinase (JNK). Taken together, these findings suggest that resveratrol ameliorates inflammation in skeletal muscle of HFD-induced model of obesity. Therefore, resveratrol might represent a potential treatment for attenuation of inflammation in skeletal muscle tissue.

## Introduction

Type 2 diabetes (T2D) has become a public healthcare problem in the recent decades and the incidence of T2D has risen in all ethnicities^1^. Insulin resistance is the hallmark of T2D etiology and it is defined as impaired response of target tissues to normal circulating levels of insulin^2^. Insulin resistance in skeletal muscle (SM) has an important role in the pathogenesis of T2D, because 80% of systemic postprandial of glucose uptake take places in this tissue^3^. Although the underlying mechanism of insulin resistance in SM is not yet clearly understood, an increased intramyocellular fat deposition and defects in mitochondrial oxidative phosphorylation have been suggested to play a major role in the progression of insulin resistance in SM^4^. Emerging evidence has also indicated that SM inflammation in obesity induces insulin resistance in this tissue^5^. Accumulating evidence reports that SM myotubes produce various numbers of cytokines in obesity state^5^. Studies have found an increased interleukin 6 (IL-6) expression in SM of individual with metabolic complications of obesity^5–7^. Myotubes from obese people generate higher levels of cytokines such as tumor necrosis factor α (TNF-α) and chemokines such as monocyte chemoattractant protein 1 (MCP-1) in comparison with myotubes from lean individuals^5,8,9^. *In vitro* studies have shown an enhanced expression of pro-inflammatory cytokines from myocytes under treatment with inflammatory cytokines and free fatty acids (FFAs)^2,10,11^. In addition to the ability of SM myocytes to secrete pro-inflammatory cytokines, the evidence demonstrates that immune cells also accumulate in SM in obesity^5^. Several reports have suggested increased accumulation of immune cells such as macrophages and T cells in SM of diabetic-obese humans and in animal models challenged with a high-fat diet (HFD)^5,12–15^. The association between immune cells accumulation and insulin resistance has been previously reported, where the absence of the F4/80^+^ cluster of differentiation (CD)11b^+^ CD11c^+^ macrophages, improved both of the SM and systemic insulin sensitivity^13^. Immune cells in SM tend to switch toward pro-inflammatory phenotypes in obesity^5^. It has been demonstrated that in obesity macrophages can switch from an anti-inflammatory M2 phenotype to a pro-inflammatory M1 phenotype^12,14–16^. In the case of T cells, an increased number of T helper1 (Th1) cells and a decreased number of regulatory T cells (Treg) has been reported for mice fed an HFD^12^.

Recently, the use of natural products derived from plants has gained considerable attention among the scientists for the prevention/treatment of numerous chronic inflammatory disorders^17^. Among many bioactive molecules derived from plants, polyphenols are of particular interest due to their potential anti-inflammatory properties^18^. There is increasing evidence that resveratrol (RES) prevents or attenuates progression of several disorders such as T2D, cardiovascular disease and cancer. The results of a meta-analysis including 11 randomized controlled trials revealed that RES intervention considerably ameliorates hyperglycemia and insulin resistance in diabetic patients^19^. There is accumulating evidence that RES inhibits the expression and secretion of pro-inflammatory mediators [such as TNF-α, IL-6, interleukin 1 beta (IL-1β), interleukin 12 and interferon γ (IFN-γ)]. Recently, a meta-analysis including 17 randomized controlled trials revealed that RES supplementation reduces plasma concentration of TNF-α and high sensitive C reactive protein (hs-CRP)^20^. Studies in animal models of obesity suggest that RES protects against adipose tissue inflammation and insulin resistance through decreasing macrophage recruitment; increasing M2 polarity cell counts and increasing the proportion of circulating Treg cells^21,22^. Despite the data on the beneficial anti-inflammatory effect of RES in several tissues^21–23^, the role of this polyphenol in control of SM inflammation in obesity remains unclear. Accordingly, we in the present study hypothesized that RES could ameliorate HFD-induced SM inflammation in mice. To this end, we investigated several markers of tissue inflammation including the expression of pro- inflammatory cytokines and chemokines, macrophage recruitment, macrophage polarity state and the frequency of T cells. In addition, we have studied the effects of RES on the mitogen-activated protein kinases (MAPKs) and adenosine monophosphate-activated protein kinase (AMPK) pathways.

## Results

### Resveratrol attenuated HFD-induced obesity

**Figure 1:**
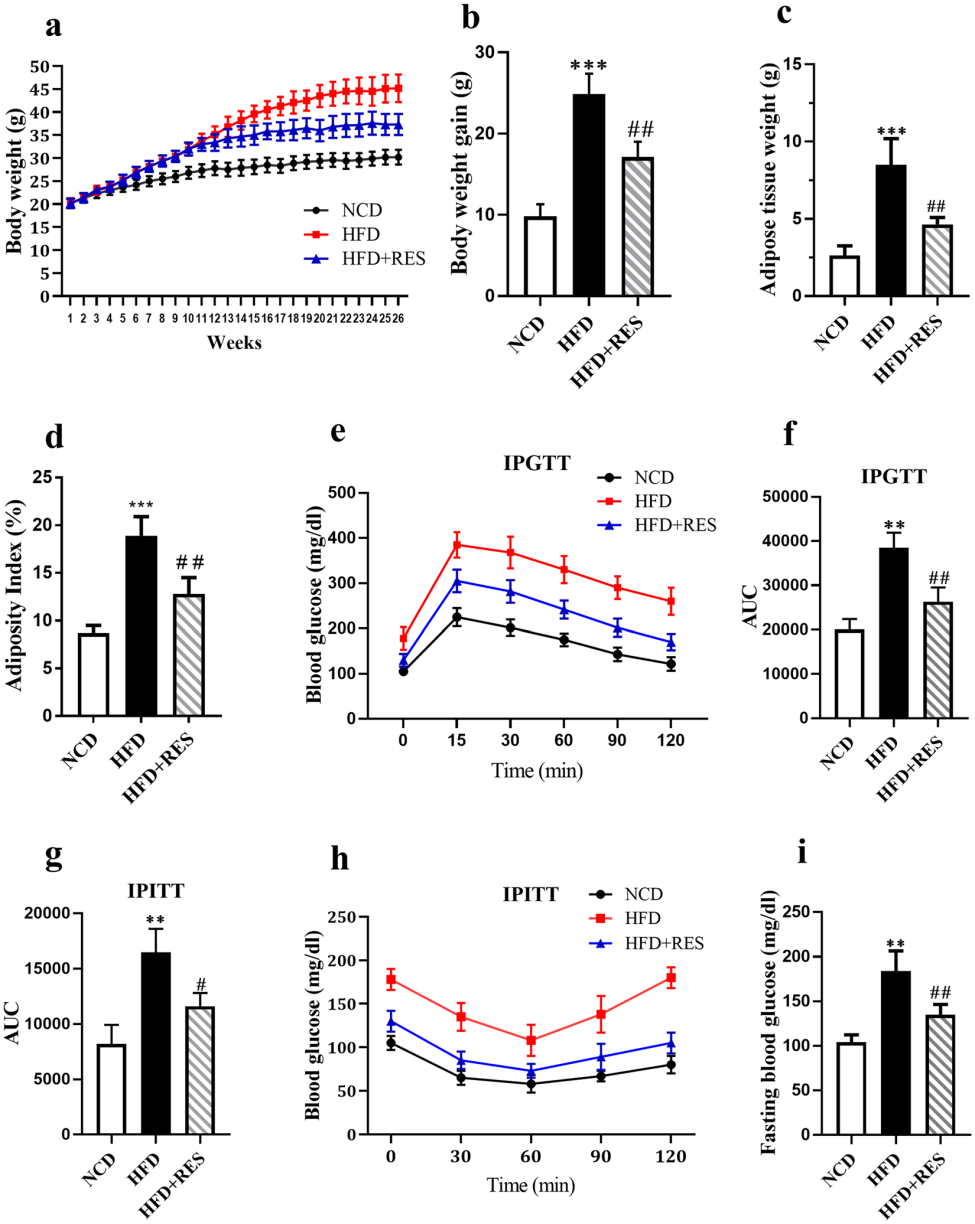
Resveratrol attenuated HFD-induced obesity. (**a**) Body weight growth curve of mice on HFD, normal chow diet (NCD) or HFD + RES diets. (**b**) Comparison of final body weight gain at the end of treatment. (**c**) Total white adipose tissues fat pads weight (**g**). (**d**) Adiposity index (%) was assessed as the white adipose tissues weight (**g**) to total body weight of mice and multiplied by 100. (**e**) Intraperitoneal glucose tolerance test (IPGTT), Blood glucose levels in HFD group (red square), NCD group (black circle) and HFD + RES group (blue triangle). (**f**) IPGTT area under the curve (AUC). (**g**) IPITT area under the curve (AUC) (**h**) Intraperitoneal insulin tolerance tests (IPITT) (n = 5). (**i**) Plasma glucose levels following a 4 hr fasting. All data were analyzed by one-way ANOVA, Tukey post-test. Values are expressed as means ± SD *p < 0.05, **p < 0.01, ***p < 0.001 vs. the control group. ^#^p < 0.05, ^##^p < 0.01, ^###^p < 0.001 vs. the HFD group. NS, not significant.

The effects of RES treatment on body weight and biochemical characteristics of the animals are illustrated in Fig. 1. As anticipated, the body weight of the animals fed on HFD gradually enhanced from fourth to tenth weeks. Resveratrol intervention for 16 weeks significantly reduced body weight gain in the HFD mice (Fig. 1a). The final average body weight gain of the control, HFD and the HFD-supplemented with 0.4% resveratrol (HFD + RES) groups were 9.8 g, 24.9 g and 15.1 g, respectively (Fig. 1b). In addition, RES treated group had a tendency to display a significant reduction in fat pad mass and the adiposity index in comparison with the HFD group (Fig. 1c,d). Animals fed the HFD displayed impairments in glucose homeostasis as evidenced by the higher glucose area under the curves (AUC) during the intra-peritoneal glucose tolerance test (ipGTT) (Fig. 1e,f), intra-peritoneal insulin tolerance test (ipITT) (Fig. 1g,h) and fasting blood glucose (Fig. 1i). However, the glucose AUCs in both ipGTT and ipITT and fasting blood glucose were significantly lower in the HFD + RES group in comparison with the HFD group. HFD and RES intervention failed to represent any evident influence on daily food intake (Data are shown in Fig. 1. of supplementary file).

### Resveratrol restricted HFD-induced macrophage accumulation in skeletal muscle tissue

**Figure 2:**
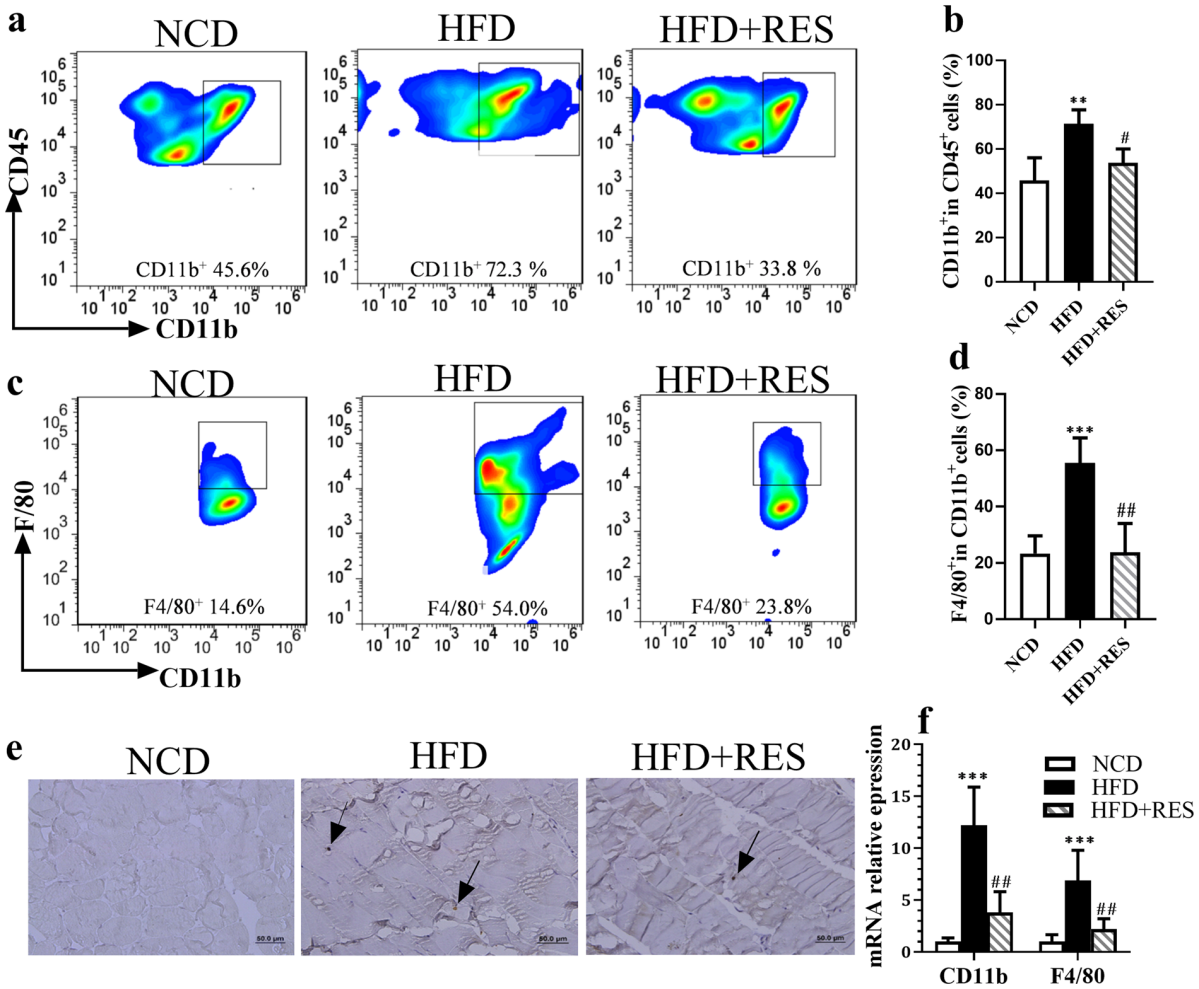
Resveratrol restricted HFD-induced macrophage accumulation in skeletal muscle tissue. (**a**) Flow cytometry plots for analysis of macrophages percentage in skeletal muscle of NCD, HFD and HFD + RES groups. (**b**) Percentage of CD11b^+^ cells in CD45 + population. (**c**) The sequential gating strategy for analysis of F4/80macrophages. (**d**) Percentage of F4/80^+^ CD11b^+^ cells (total macrophages). (**e**) F4/80 Immunohistostaining in skeletal muscle of the experimental groups. Arrows indicate the macrophages presence between the muscle fibers, Scale bar: 50 μm. (**f**) CD11b and F4/80 mRNA relative expression to control in SM tissue by RT-PCR. All data were analyzed by one-way ANOVA, Tukey post-test. Values are expressed as means ± SD *p < 0.05, **p < 0.01, ***p < 0.001 vs. the control group. ^#^p < 0.05, ^##^p < 0.01, ^###^p < 0.001 vs. the HFD group.

Recent findings suggest that meta-inflammation has been involved in the etiology of insulin resistance in SM. To gain insight into whether RES treatment diminishes inflammation in SM tissue, we evaluated the percentage of macrophage infiltration in this tissue by flow cytometry using cluster of differentiation (CD) 45 as a myeloid marker and CD11b and F4/80 as macrophage markers. Flow cytometric analysis revealed that HFD loading increased the percentage of CD11b^+^ cells within the CD45 positive cell population, compared with mice fed the normal chow diet (NCD), whereas the percentage of CD11b^+^ cells was significantly lower in the HFD + RES group in comparison with the HFD group. (Fig. 2a,b). The proportion of F4/80^+^ within the CD11b^+^ cell population, was notably higher in the HFD group than in control mice. Importantly a reduction in the percentage of F4/80 in HFD + RES mice was observed in comparison with the HFD-fed mice (Fig. 2c,d). Also the recruitment of macrophages into SM tissue was confirmed by immunohistochemical staining for rodent macrophage marker (F4/80). As shown in Fig. 2e, albeit HFD-fed mice had strongly positive intense staining of F4/80, but HFD + RES mice had lower positive staining, suggesting a decreased presence of the macrophages in SM tissue (Fig. 2e). These findings were further supported by gene expression analyses. The results from real-time polymerase chain reaction (RT-PCR) experiments suggested that HFD feeding elevated the mRNA levels of CD11b^+^ and F4/80^+^ in SM tissue compared with NCD-fed mice and these findings were apparently blunted by RES administration (Fig. 2f).

### Resveratrol switched M1 to M2 macrophages

**Figure 3:**
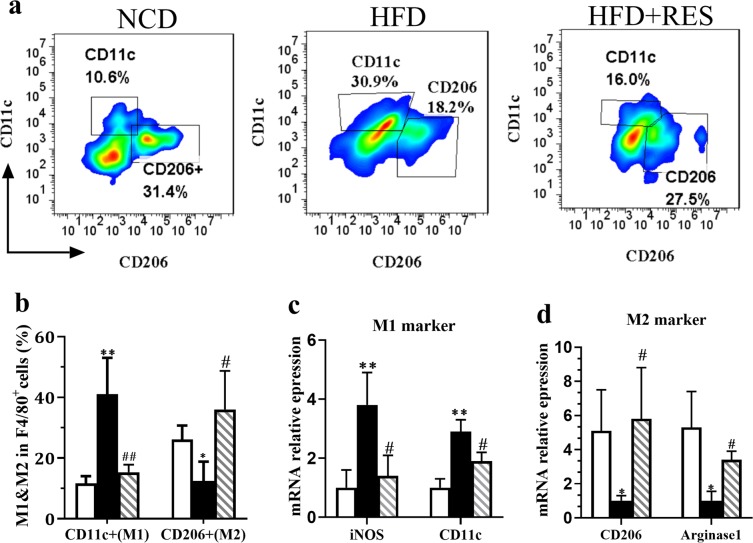
Resveratrol switched M1 to M2 macrophages. (**a**) Representative flow cytometry plots and the sequential gating strategy for CD11c and CD206 markers in CD11b and F4/80 dual positive cells for analysis of macrophages polarization in skeletal muscle tissue of NCD, HFD and HFD + RES groups. (**b**) Percentage of CD11c^+^ cells and CD206^+^ cells in total macrophages. (**c**) iNOS and CD11c (M1 markers) mRNA expression in skeletal muscle tissue. (**d**) CD206 and arginase1 (M2 markers) mRNA expression in skeletal muscle tissue. All data were analyzed by one-way ANOVA, Tukey post-test Values are expressed as means ± SD *p < 0.05, **p < 0.01, ***p < 0.001 vs. the control group. ^#^p < 0.05, ^##^p < 0.01, ^###^p < 0.001 vs. the HFD group.

The heterogeneity of macrophage phenotypes has been shown from various human tissues^24–26^. Macrophages show a dynamic transition between two polarization state, M1 (pro-inflammatory) and M2 (anti-inflammatory)^27,28^. In the current study, we investigated whether RES treatment could affect the relative frequency of M1/M2 macrophages in SM tissue using flow cytometry via staining with anti-CD11c, cell surface markers for M1 and anti-CD206, intracellular marker for M2 macrophages. By gating of dual-positive CD11b^+^ F4/80^**+**^ cells, it was found that HFD feeding resulted in a dramatically diminution of M2 cells subset (CD206^+^, CD11c^−^) (12%) and increase of the percentage of M1 cells subset (CD11^+^, CD206^−^) (40%) compared to control mice (Fig. 3a,b). Importantly, RES switched M1 to M2 macrophages in SM of mice fed HFD. We further confirmed above data by evaluating the expression of the genes involved in macrophage heterogeneity. While, HFD feeding decreased the expression of M2 phenotype markers (Arginase and CD206) and increased M1 phenotype markers [inducible nitric oxide synthase (iNOS) and CD11c], RES treatment could significantly reverse these effects in HFD-treated mice (Fig. 3c,d). These findings indicated the potency of RES in ameliorating inflammation in SM tissue through the inhibition of M1 and the promotion M2 macrophages polarization.

### Resveratrol enhanced the percentage of T regulatory cells

**Figure 4:**
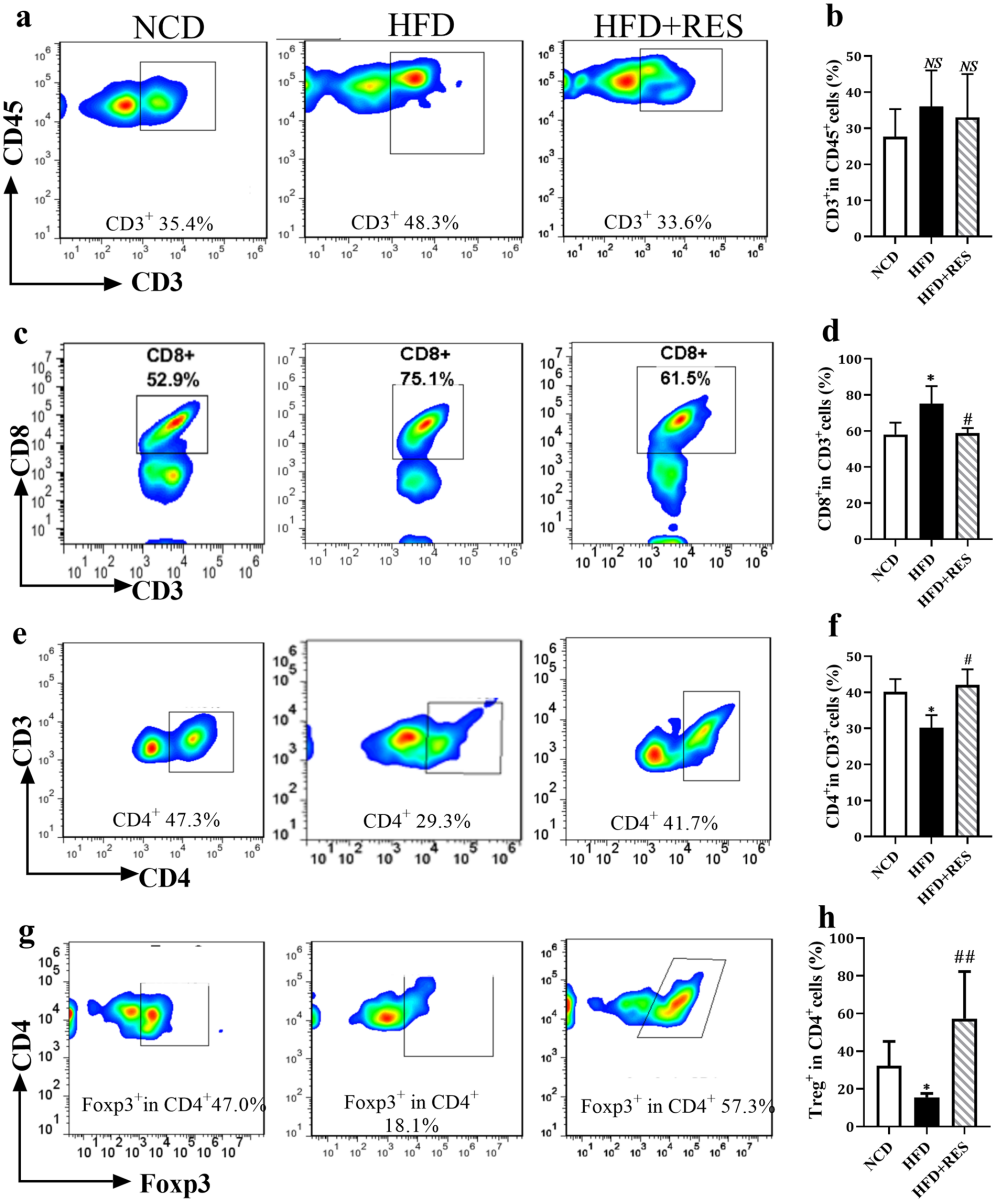
Resveratrol enhanced the percentage of T regulatory cells. (**a**) Representative flow cytometry plots and the sequential gating strategy for analysis of T cells in skeletal muscle tissue. (**b**) Percentage of CD3^+^ cells (T cells). (**c**) Sequential gating strategy for analysis of CD8^+^ cells in skeletal muscle. (**d**) Percentage of CD8^+^ cells. (**e**) Sequential gating strategy from CD3^+^ cells for analysis of CD4^+^ cells in skeletal muscle. (**f**) Percentage of CD4^+^ cells. (**g**) Sequential gating strategy for analysis of Treg cells in skeletal muscle. (**h**) Percentage of FOXP3^+^ CD4^+^ cells (Treg cells) in skeletal muscle tissue. All data were analyzed by one-way ANOVA, Tukey post-test. Values are expressed as means ± SD *p < 0.05, **p < 0.01, ***p < 0.001 vs. the control group. ^#^p < 0.05, ^##^p < 0.01, ^###^p < 0.001 vs. the HFD group, NS = no statistically significant difference between groups.

Apart from macrophages, lymphocytes appear to be strongly involved in inflammatory processes^24^. Helper T cells (CD4^+^ T cells), cytotoxic T cells (CD8^+^ T cells) and regulatory T cells (Treg cells) are main subsets of T lymphocytes^29^. To investigate the effects of RES on lymphocytes, we first evaluated CD3, a T cell co-receptor that helps to activate both the cytotoxic T cells and T helper cells. No significant difference in T cell marker of CD3 was detected between three groups (Fig. 4a,b). An elevation of CD8^+^ T cells was observed in HFD mice, whereas RES significantly prevented the increase of these cells in the SM tissue of HFD group (Fig. 4c,d). We observed a reduction in the percentage of CD4^+^ cells in the HFD groups compared to those in the NCD group, however resveratrol intervention significantly increased the percentage of CD4^+^ cells in SM of obese mice (Fig. 4e,f). Feeding the HFD was followed by a significant reduction in the frequency of forkhead box protein3 (Foxp3)^+^ CD4^+^ regulatory T (Treg) cells in SM, whereas an enhanced percentage of Treg cells was found in response to RES administration (Fig. 4g,h).

### Resveratrol down regulated TLR cascade in skeletal muscle

**Figure 5:**
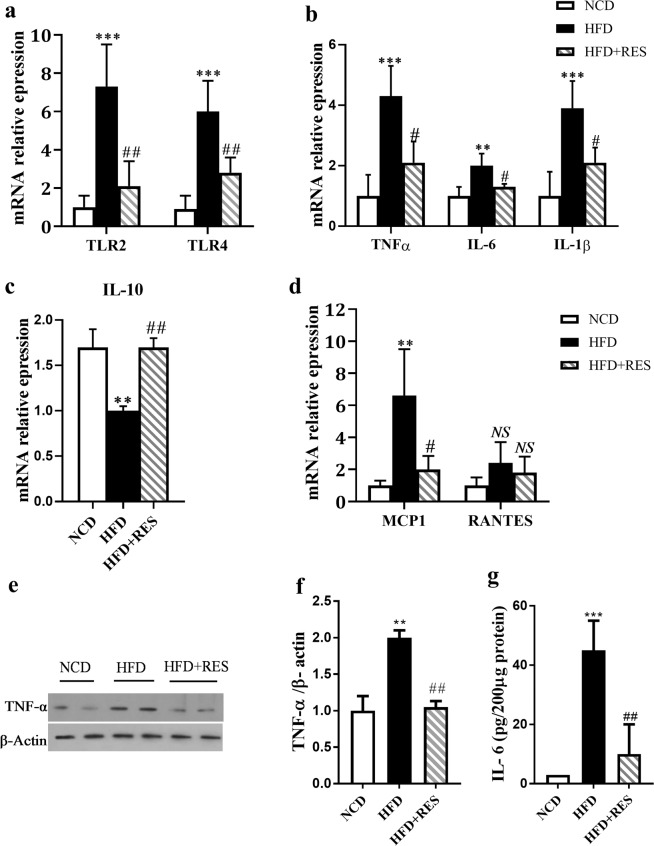
Resveratrol down regulated TLR cascade in skeletal muscle. (**a**) Relative expression of TLR2 and TLR4 were determined in skeletal muscle by RT-PCR. (**b**) Relative expression of TNF-α, IL-6 and IL-1β in skeletal muscle. (**c**) Relative expression of IL-10 (**d**) relative expression of MCP-1 and RANTES in skeletal muscle tissue. (**e**,**f**) Western blot analysis of TNF-α protein level. (**g**) The protein level of IL-6 was assessed by ELISA. All data were analyzed by one-way ANOVA, Tukey post-test. Data are presented as means ± SD *p < 0.05, **p < 0.01, ***p < 0.001 vs. the control group. ^#^p < 0.05, ^##^p < 0.01, ^###^p < 0.001 vs. the HFD group, NS = no statistically significant difference between groups.

Toll-like receptors (TLRs) participate in the initiation of the inflammatory processes^30,31^. TLR-2 and TLR-4 activation leads to inflammatory cytokine production such as TNF-α and IL-6^32–34^. The results of the current study revealed that the expression of TLR-2 and TLR-4 were higher in SM of HFD group compared to NCD group. Resveratrol significantly reduced the expression level of these genes in HFD treated mice (Fig. 5a). To further understand the influence of RES on inflammatory responses, we measured the expression of several cytokines. The expression of pro-inflammatory cytokines in SM tissue indicated a significant decrease for IL-6, TNF-α and IL-1β in the HFD + RES group in comparison with HFD group (Fig. 5b). The mRNA data of IL-6 and TNF-α were further confirmed at protein levels (Fig. 5e–g). Moreover, a significant increase in interleukin 10 (IL-10) expression was found in SM of NCD-fed mice compared to animal fed an HFD alone (Fig. 5c). To understand the underlying mechanism of decreased macrophage recruitment to SM tissue, we assessed the expression of MCP-1, an important chemokines that regulate migration and infiltration of monocytes/macrophages^33,35^. We found that the expression of MCP-1 was statistically increased in the HFD groups compared to those in the NCD group. Resveratrol administration significantly decreased the expression level of MCP-1 in SM compared to that in the HFD mice (Fig. 5d). We also evaluated the expression of regulated upon activation normal T cell expressed and secreted (RANTES) or cc-chemokine ligand 5 (CCL5), a chemokine which is involved in recruitment of T cells^36^. There were no statistically significant differences in the expression of RANTES between three groups (Fig. 5d).

### Effect of resveratrol on skeletal muscle lipid content

**Figure 6:**
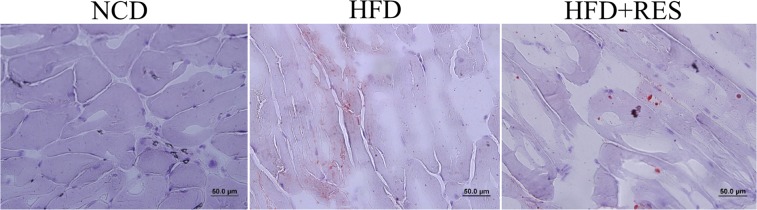
Representative sections of gastrocnemius stained with Oil Red O. Red indicates the intramascular lipid deposition.

Increased intra-myocellular lipid deposition has been shown to be correlated with diminished insulin sensitivity and increased recruitment of macrophages in SM^37^. To assess lipid deposition in this study muscle sections were stained by Oil‐Red O. An increased intramuscular lipid area could be observed in HFD animals, whereas HFD + RES had the opposite effect (Fig. 6).

### Resveratrol attenuated HFD-induced inflammation through inhibiting the MAPKs pathway

**Figure 7:**
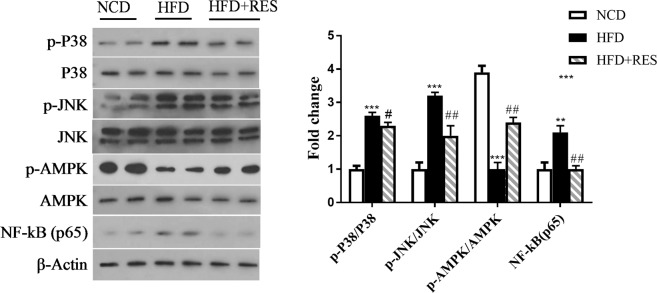
Representative western blot is shown. Phosphorylated p38, total p38, phosphorylated JNK, total JNK, phosphorylated AMPK, total AMPK and NF-κB (p65), and control β-Actin levels were evaluated by immunoblot analysis. Protein bands were quantified by densitometry and normalized to β-Actin levels. Full-length blots are presented in Supplementary Fig. 3. All data were analyzed by one-way ANOVA, Tukey post-test. Data are presented as means ± SD *p < 0.05, **p < 0.01, ***p < 0.001 vs. the control group. ^#^p < 0.05, ^##^p < 0.01, ^###^p < 0.001 vs. the HFD group.

Inflammatory responses are regulated by several signaling pathways including the AMPK and MAPKs. It has been suggested that elevation of MAPKs phosphorylation shifts the cellular environment to a more inflammatory state^27,33,38^. We in this study aimed to investigate the mechanisms underlying the anti-inflammatory effect of RES by evaluating the AMPK and MAPKs pathways. Our findings indicated that phospho-p38, phospho-c-Jun N-terminal kinase (JNK) and nuclear factor kappa-light-chain-enhancer of activated B cells (NF-κB) (p65) levels were upregulated in SM of the HFD group, whereas, these findings were markedly lower in RES-treated mice. We also observed that phospho-AMPK tended to be suppressed by HFD, whereas RES attenuated HFD-suppressed phospho-AMPK in SM of HFD-fed mice (Fig. 7).

## Discussion

Previous studies have suggested that lowering inflammation could be an effective approach in prevention/treatment of obesity–related disease^1,39,40^. In this context, RES was suggested to attenuate inflammation in several tissues^2,41–43^, however, the role of this polyphenol in SM inflammation *in vivo* remains unclear. Our data in this study revealed that resveratrol has a potent anti-inflammatory effect in SM of HFD-fed model of obesity.

We first evaluated our model of HFD-induced obesity. The significant difference in body weight gain among HFD and HFD + RES groups represented that RES has a desirable efficacy in mitigation of the body weight gain without any influence on daily energy intake. Resveratrol improved glucose tolerance and insulin sensitivity in HFD-fed mice and these findings were in agreement with kim *et al*. and Lagouge *et al*. studies^44,45^.

In this study, we gathered the data from a set of inflammatory markers. Macrophages play a central role in obesity-induced inflammation and accumulation of these cells in metabolic tissues has been reported to have important roles in the pathophysiology of several metabolic disorders such as chronic liver disease, atherosclerosis, and diabetes^46^. Furthermore, we evaluated macrophage heterogeneity that is the important part of the tissue inflammation. We also investigated more particularly the relationship between RES and inflammation in SM by evaluating the expression level of several pro-inflammatory and anti-inflammatory cytokines and chemokines. This set of inflammatory markers has improved our understanding on the possible effect of RES on SM inflammation in our model of obesity. The data of this study suggested that RES treatment has the beneficial anti-inflammatory effects on SM of HFD fed mice. More specifically, investigation of macrophage infiltration in SM by three methods, real-time PCR, immunohistochemistry and flow cytometry, strongly demonstrated a decrease in transcript and protein levels of the macrophage-specific markers CD11b and F4/80 in the SM of HFD-RES fed mice, indicating that RES prevented macrophage accumulation in SM tissue. While about 60% of the leukocytes in the SM following HFD feeding were CD11b^+^ F4/80^+^ macrophages, this number declined to 20% in RES treated mice. In support of these data, we also found that RES reduced the expression of MCP-1, a chemokine that recruits monocytes toward the tissues^13^. Also our findings revealed that RES treatment reduced M1 polarization and promoted M2 polarization of the macrophages. This finding was further confirmed by real-time PCR. Increased expression of M1 markers such as CD11c, and iNOS and decreased expression of CD206 and Arginase, M2 markers, under HFD challenge were reversed by RES. Finally, above findings were in consistent with lower expression levels of pro-inflammatory cytokines (TNF-α, IL-1β and IL-6) and higher expression of the anti-inflammatory cytokine (IL-10) following RES treatment in SM of HFD fed mice. In support of above mentioned findings, several studies have shown the beneficial anti-inflammatory effect of RES in various tissues, but not in SM tissue. Jeon *et al*. reported that RES reduced macrophage infiltration to adipose tissue in HFD-fed animals^22^. Intermittent hypoxia caused increase in total number of macrophages in visceral adipose tissue which consists of increase in the pro-inflammatory M1 macrophage and reduction in M2 macrophages. These changes were markedly abrogated in RES administration state^21^. In Jeong *et al*. study the expression of F4/80 in the liver was statistically lower in the RES group than in the HFD fed animals^47^. Resveratrol administration suppressed Kupffer cells recruitment and down-regulated the expression of pro-inflammatory cytokines such as TNF-α and IL-6 in bile duct ligation and carbon tetrachloride (CCl_4_)-induced liver injuries models^23,48^. In Yong *et al*. study the microglia polarization state was modulated by the RES administration in LPS-induced neuroinflammation and RES switched the microglia to M2 phenotype^49^. Buttari *et al*. also demonstrated that RES reduces inflammation, M1 macrophage accumulation and expression of cytokines in response to 7-Oxo-Cholestrol in human monocyte-derived macrophages^50^ In addition, it has been reported that RES treatment significantly inhibited palmitate-induced inflammation in C2C12 cells^2^. Taken together, the data from the present study provide the evidence that RES attenuates SM inflammation in HFD fed model of obesity through reducing macrophage recruitment, increasing M2 polarity cell counts and subsequently decreasing the expression and release of pro-inflammatory cytokines.

Growing evidence indicates an increased T cell numbers in SM of obese people with abnormal glucose tolerance or T2D^5,12,14^. In addition, an enhanced accumulation of T cells in the SM tissue of HFD-fed mice was reported^5,12,14^. In the current study, the percentage of CD3^+^ cells in SM did not differ between the experimental groups and this finding was supported by no change in the expression of RANTES in SM tissue. These data are in agreement with Carreras *et al*. study reports no change in the number of CD3^+^ lymphocytes in visceral adipose tissue^21^, however, this outcome is not in accordance with the results of the studies by Khan *et al*.^12^ and Fink *et al*.^14^. Khan *et al*. observed an apparent increase in T cells count in SM and para-muscular adipose tissue of HFD feeding mice, while Fink *et al*. reported increase in T cells count in quadriceps muscle only. The discrepancy between the studies might be due to the selection of the different type of muscle. Our finding of increased CD8 and decreased Treg cells percentage in HFD group were in the line with previous studies. It has been shown that CD8^+^ T cell numbers and Treg cells within the CD4^+^ T cell population were increased and decreased, respectively in SM of obese mice^12^. In adipose tissue of obese mice, an increased CD8^+^ cells and reduced regulatory T cells was reported^51^. Importantly, we observed that RES treatment significantly reduced the percentage of CD8^+^ cells and increased the percentage of Treg cells in SM tissue of the HFD fed mice. In line with this finding, RES was reported to increase the amount of T regulatory cells through activation of the aryl hydrocarbon receptor in an animal model of obesity^52^. In addition, RES supplementation enhanced the percentages of CD3^+^ CD4^+^/CD3^+^ CD8^+^ subsets when challenged with an HFD^52^.

Studies have demonstrated that SM insulin resistance is linked to with elevated amount of circulating FFAs and triglycerides and enhanced intramyocellular deposition of lipid intermediates^53,54^. The ectopic deposition of these lipid intermediates has been associated with impairments in the insulin signaling cascade^54^. In addition to the potential direct effect on the insulin signaling, increased muscle lipid deposition has been reported to induce muscle inflammation. In this regard, palmitic acid and its metabolite ceramide have been found to activate pro-inflammatory signaling through the TLR-2/4 pathway^55^. In the present study, we found that increased fat deposition following HFD feeding in SM is correlated with upregulation of TLR-2 and TLR-4 pathway. Importantly, RES prevented HFD-induced lipid accumulation and TLR-2 and TLR-4 expression in SM tissue. These findings suggest that RES treatment led to less lipid content and subsequently lower activation of the TLR signaling in muscle tissue. These findings along with lower body weight of the mice following RES treatment suggest the possibility of an indirect inhibitory effect of RES on muscle inflammation through reducing body weight or fat mass.

The studies have demonstrated that obesity-induced inflammation was negatively correlated with AMPK activation^56,57^. It was suggested that a lower level of AMPK activity could be responsible for enhanced inflammation in the visceral adipose tissue and systemic insulin resistance in diabetic-obese individuals^57,58^. Furthermore, lipopolysaccharide, FFAs and diet-induced obesity reduced phosphorylation of the AMPK in the adipose tissue and macrophages^59^. Several studies using multiple cell lines have suggested that AMPK prevents inflammatory responses via indirectly inhibiting nuclear factor kappa-light-chain-enhancer of activated B cells (NF-κB)^60^. To investigate whether the inhibitory effects of RES on inflammation are mediated through the regulation of the AMPK, we evaluated the phosphorylation of the AMPK in SM tissue. In the present study reduced phosphorylation of AMPK following HFD feeding was evidently enhanced in RES–treated mice. Given the anti-inflammatory property of the AMPK activation, it is plausible to suggest that RES inhibitory effects on SM inflammation are at least partly mediated through the activation of the AMPK.

MAPKs are the molecular links between obesity and inflammation. JNK and p38, members of the MAPK family can be activated by TNF-α, IL-1β, endoplasmic reticulum (ER) stress, and saturated FFAs^2,5,61,62^. Several investigations have demonstrated that the activation of MAPKs is important in regulation of inflammation via controlling the activation of NF-κB and inhibitor of kappa B (IκB) kinases (IKKs)^63–65^. To determine the molecular mechanisms underlying the anti-inflammatory properties of resveratrol, we targeted the MAPKs pathway. We found that HFD treatment resulted in a robust increase in JNK and p38 phosphorylation and enhance NF-κB level in SM, whereas these effects were markedly blunted by RES. These findings are in the line with the data from different cell types such as microglial^66^, U937^67^ and macrophages^68^.

In summary, the data of the present study provided the evidence that RES markedly attenuated SM inflammation in HFD fed mice. Resveratrol ameliorated inflammation through decreasing macrophage recruitment, increasing M2 polarity cell counts, inducing the proportion of Treg cells, reducing the population of M1 polarity and subsequently decreasing the expression of the pro-inflammatory cytokines in SM of HFD-fed mice. It appears that the anti-inflammatory effects of RES in SM are mediated through activation of the AMPK and inhibition of the MAPKs pathways. Taken together, these findings suggest that RES might represent a potential treatment for attenuation of inflammation in SM tissue.

## Methods

### Experimental design

All experiments in this study were carried out equally with protocols approved by the Research Committee and the Medical Ethics and History of Medicine Research Center at Tehran University of Medical Sciences. Thirty 6-week-old male C57/BL6 mice were obtained from Pasteur Institute of Iran. Mice were individually housed under standard conditions of illumination and ambient temperature. After an acclimatization period, the animals were randomly categorized into two dietary groups for 10 weeks ; normal chow diet [NCD, 10 kcal% fat, n = 10] and a high fat diet [HFD, 55.9 kcal% fat, n = 20]. After this period, half of the HFD-fed mice were fed with HFD-supplemented with 0.4% Resveratrol (4 g/kg diet) [HFD + RES] for 16 weeks. Throughout the duration of the experiment, the body weight was recorded weekly. At the end of the study, intra-peritoneal glucose tolerance test (ipGTT) and intra-peritoneal insulin tolerance test (ipITT) were performed^69^. After 26 weeks, mice were sacrificed by intraperitoneally injection of ketamine-xylazine anesthesia. The gastrocnemius muscle was rapidly excised, weighed and rinsed in cold phosphate-buffered saline (PBS) to remove excess blood thoroughly and immediately used for flow cytometry analysis. A portion of the other side of the gastrocnemius muscle tissues were snap frozen in liquid nitrogen and stored at −80 °C for gene analysis and western blot. Another portion of the SM was fixed in 10% (v/v) neutral buffered formalin (NBF) for immunohistochemistry and hematoxylin and eosin (H&E) staining. All experiments were performed in compliance with relevant guidelines and regulations.

### ipGTT and ipITT

ipGTT was performed following a 6 h fast, mice were administered with glucose (1 g/kg) via intraperitoneally (ip) injection. Blood samples were taken from the tail vein at 0, 15, 30, 60 and 120 minutes and measured using an Accu check Aviva blood glucose monitor (Roche Diagnostics, Burgess Hill, UK). For the ipITT, a dose of 0.75 U/kg of regular human insulin was administered intraperitoneally in 4-hour fasted mice. Blood glucose concentrations were measured using the same glucometer as above. Area under the curve (AUC) for the ipGTT and ipITT were measured starting at baseline (0 min) values.

### Gene expression analysis

Total RNA from muscle tissues was extracted using GeneAll Hybrid-R RNA purification kit. Complementary DNA (cDNA) was made in reverse transcription reaction using a RevertAid First Strand cDNA Synthesis Kit (Thermo Fisher Scientific). The relative expression of the selected genes was determined by real-time quantitative polymerase chain reaction (RT-qPCR) using SYBR Green RealQ Plus 2x Master Mix Green (Ampliqon). Quantitative PCR was run on StepOnePlus™ Real- Time PCR System. The results were normalized to β-actin expression level and 2^−ΔCT^ was used to compare the relative expression of target genes between groups. All primer sequences used in quantitative PCR analysis are listed in Table S1 of the Supplementary File.

### Flow cytometry

Skeletal muscle tissues were minced and digested with type B collagenase (2 mg/ml, Sigma-Aldrich, Germany) and type D collagenase (1 mg/ml, Sigma-Aldrich, Germany) in high glucose Dulbecco’s modified eagle’s medium (DMEM) at 37 °C for 45 min with gentle agitation. Cell suspensions were filtered through a 40μm cell strainer and centrifuged at 500 g for 5 min at 4 °C. After washing the cells with PBS, pellets were suspended in cold fluorescence-activated cell sorting (FACS) buffer, pre-incubated with mouse Fc receptor-blocking antibodies (anti-CD16/CD32) (BD Pharmingen, San Diego, CA, USA) on ice for 15 min. Cells were stained with fluorophore-conjugated antibodies for macrophage markers: Cluster of Differentiation 45 (CD45) (Bio legend, San Diego, CA, USA), CD11b (Biolegend, San Diego, CA, USA), F4/80 (eBioscience, San Diego, CA, USA), CD11c (BD Pharmingen San Diego, CA, USA) and T cells markers: CD45, CD3e (Bio legend San Diego, CA, USA), CD8 (Bio legend, San Diego, CA, USA), CD4 and CD25 (eBioscience, San Diego, CA, USA). To detect CD206 marker (Biolegend, San Diego, CA, USA) and Treg cells (eBioscience, San Diego, CA, USA) intracellular staining was performed according to the manufacturer’s recommendations. Finally, the cells were resuspended in cold FACS buffer, and flow cytometry analysis was performed using an Attune NxT flow cytometer (Thermo Fisher). Data were analyzed using FlowJo software. The antibodies used in this study are listed in Table S2 of the Supplementary File.

### Immunohistochemical staining

5 µm sections paraffin-embedded gastrocnemius tissues were deparaffinized and rehydrated. For antigen retrieval, the sections were boiled in citrate buffer (10 mM, pH 6.0) for 20 min. The sections were then incubated in 3%H_2_O_2_ for 10 min at room temperature for quenching of endogenous peroxidase. The slides were blocked in TBST (Tris-buffered saline (TBS) with 0.5% Tween-20) with 5% normal goat serum for 1 h at room temperature. Incubation of primary antibody against F4/80 (Bio-Rad, San Diego, CA, USA) was performed overnight at 4 °C in a humidity chamber. After washing with TBST, the sections were incubated with horseradish peroxidase (HRP) goat anti-rat IgG secondary antibody (AP136P, Sigmaaldrich, Germany) for 30 min at room temperature. The sections were visualized by high quality diaminobenzidine (DAB) substrate and then hematoxylin counter stain was applied.

### Oil red O staining

After dissection, muscle tissues were embedded in optimal cutting temperature medium (OCT) (Bio-optica, Milano, Italy) and immediately frozen at −80 °C. Frozen tissues were cryostat sectioned. After immersing in 60% isopropanol for 5 min, sections were stained with freshly filtered Oil Red O solution for 45 min at room temperature. The sections were washed with 60% isopropanol and then distilled water. The cryosections were then stained with hematoxylin.

### Western blotting analysis

Muscles tissues homogenates were prepared in cold radioimmunoprecipitation assay (RIPA) buffer. Protein concentration were determined using a bicinchoninic acid (BCA) protein assay kit (ThermoFisher Scientific). Equal amounts of protein were separated on 10% sodium dodecyl-sulfate polyacrylamide gel electrophoresis (SDS-PAGE). After transferring the bands onto polyvinylidene difluride (PVDF) membrane, the membrane was blocked with 5% bovine serum albumin (BSA) in TBST for 2 h at room temperature. Incubation of membrane with primary antibodies were performed overnight at 4 °C. After 30 min washing in TBST solution, membranes were incubated with HPR-coupled secondary antibody for an hour at room temperature. Labeled proteins were visualized using enhanced chemiluminescent substrate (ECL, Amersham). The intensity of bands was determined by densitometry with the Image J software. The antibodies used in this study are listed in Table S3 of the Supplementary File.

### IL-6 measurements

The expression of IL-6 protein in SM homogenates was measured using Mouse IL-6 ELISA Ready-Set-Go kit (eBiosciences, San Diego, CA), following the manufacture’s recommendations.

### Statistics

All statistical analyses were performed with statistical package for the social sciences (SPSS) 20. (SPSS, Chicago, IL, USA). Data are represented as means ± Standard deviation (SD). Data were analyzed by one way analysis of variance (ANOVA) test followed by Tukey post hoc tests. Values of p < 0.05 were accepted statistically significant difference. Graphs were prepared using GraphPad Prism version 7.

## Supplementary information

Supplementary information.

## Data Availability

Data available on request from the authors.
