# Supplementary material for: Resveratrol alleviates obesity-induced skeletal muscle inflammation via decreasing M1 macrophage polarization and increasing the regulatory T cell population
Source: Sci Rep. 2020 Mar 2;10:3791. doi: 10.1038/s41598-020-60185-1 (PMC7052230; doi:10.1038/s41598-020-60185-1)

**Resveratrol alleviates obesity-induced skeletal muscle inflammation via decreasing M1 macrophage polarization and increasing the regulatory T cell population**

Maryam Shabani^1^, Asie Sadeghi ^2^, Hossein Hosseini^1,^ Maryam Teimouri ^1^, Reyhaneh Babaei Khorzoughi^1,^ Parvin Pasalar ^1^, Reza Meshkani ^1*^

1. Department of Clinical Biochemistry, Faculty of Medicine, Tehran University of Medical Sciences, Tehran, I.R Iran.

2-Department of Clinical Biochemistry, Afzalipour School of Medicine, Kerman University of Medical Sciences, Kerman, Iran*.*

Running Title: Resveratrol attenuates skeletal muscle inflammation

Supplementary Table S1: Primers were used in this study

| Item | **Primer’s name** | **Sequence (5'->3')** | **Primer length** | **Product length** |
| --- | --- | --- | --- | --- |
|  |  |  |  |  |
| 1 | Mouse β-ACTIN Forward | CATCCGTAAAGACCTCTATGCCAAC | 25 | 171 |
|  | Mouse β-ACTIN Reverse | ATGGAGCCACCGATCCACA | 19 |  |
| 2 | Mouse iNOS Forward | TCCTACACCACACCAAAC | 18 | 199 |
|  | Mouse iNOS Reverse | CTCCAATCTCTGCCTATCC | 19 |  |
| 3 | Mouse CD11b Forward | AGACGTGAATGGGGACAAAC | 20 | 106 |
|  | Mouse CD11b Reverse | GAGGCTGGCTATTGATGCTC | 20 |  |
| 4 | Mouse F4/80 Forward | TGACTCACCTTGTGGTCCTAA | 21 | 111 |
|  | Mouse F4/80 Reverse | CTTCCCAGAATCCAGTCTTTCC | 22 |  |
| 5 | Mouse CD 206 Forward | CCTCTGGTGAACGGAATGAT | 20 | 161 |
|  | Mouse CD 206 Reverse | CTTCCTTTGGTCAGCTTTGG | 20 |  |
| 6 | Mouse Arginase1 Forward | TTGGCTTGCTTCGGAACTC | 19 | 148 |
|  | Mouse Arginase1 Reverse | GGAGGAGAAGGCGTTTGC | 18 |  |
| 7 | Mouse MCP1 Forward | ACTGCATCTGCCCTAAGGTCTTCA | 24 | 105 |
|  | Mouse MCP1 Reverse | AGAAGTGCTTGAGGTGGTTGTGGA | 24 |  |
| 8 | Mouse RANTES (CCl5) Forward | ATCTTGCAGTCGTGTTTGTCA | 21 | 74 |
|  | Mouse RANTES (CCl5) Reverse | TTCTTGAACCCACTTCTTCTCTG | 23 |  |
| 9 | Mouse TLR2 Forward | GCATCCGAATTGCATCACCG | 20 | 70 |
|  | Mouse TLR2 Reverse | CCTCTGAGATTTGACGCTTTGT | 22 |  |
| 10 | Mouse TLR4 Forward | TCCCTGCATAGAGGTAGTTCC | 21 | 119 |
|  | Mouse TLR4 Reverse | TCAAGGGGTTGAAGCTCAGA | 20 |  |
| 11 | Mouse IL-10 Forward | ATGCTGCCTGCTCTTACTGACTG | 23 | 216 |
|  | Mouse IL-10 Reverse | CCCAAGTAACCCTTAAAGTCCTGC | 24 |  |
| 12 | Mouse IL-6 Forward | GTTCTCTGGGAAATCGTGGA | 20 | 138 |
|  | Mouse IL-6 Reverse | TCCAGTTTGGTAGCATCCATC | 21 |  |
| 13 | Mouse IL-1β Forward | CCTTCCAGGATGAGGACATGA | 21 | 71 |
|  | Mouse IL-1β Reverse | TGAGTCACAGAGGATGGGCTC | 21 |  |
| 14 | Mouse TNF-α Forward | TGCTCTGTGAAGGGAATGGG | 20 | 142 |
|  | Mouse TNF-α Reverse | ACCCTGAGCCATAATCCCCT | 20 |  |
| 15 | Mouse CD11c Forward | GCAGAGCCAGAACTTCCCAA | 20 | 86 |
|  | Mouse CD11c Reverse | TGCTACCCGAGCCATCAATC | 20 |  |

Supplementary Table S 2: Fluorochrome antibodies were used in this study

| **Target** | **Fluorochrome** | **Clone** | **Catalog No.** | **Company** |
| --- | --- | --- | --- | --- |
| CD45 | APC/cy7 | [30-F11](https://www.biolegend.com/en-us/search-results?Clone=30-F11) | 103115-25μg | Biolegend |
| CD45 | APC | 30-F11 | 103111-25μg | Biolegend |
| CD11b | PerCP/cy5.5 | M1/70 | 550993 | BD Pharmingen |
| CD11c | PE | HL3 | 561044 | BD Pharmingen |
| F4/80 | Alexa Fluor-488 | BM8 | 53-4801-80 | eBiosciences |
| CD206 | Alexa Fluor-647 | C068C2 | 141711 | Biolegend |
| CD3ε | Percp/cy5.5 | H57-597 | 100327-25 | Biolegend |
| CD8a | PE | 53-6.7 | 100707-50 | Biolegend |
| CD4 | FITC | RM4-5 | 88-8118-40 | eBiosciences |
| CD25 | PE | PC61.5 | 88-8118-40 | eBiosciences |
| Foxp3 | APC | FJK-16S | 88-8118-40 | eBiosciences |
| CD16/CD32 | - |  | 88-8118-40 | eBiosciences |
| * AF=AlexaFluor, APC=Allophycocyanin, PE= Phycoerythrin, FITC=Fluorescein isothiocyanate, PerCP=Peridinin Chlorophyll Protein Complex, Cy=Cyanine, | | | | |
| Rat IgG2a Isotype Control APC | | | | |
| Anti-Mouse CD16/32 Purified (BD Pharmingen)  Supplementary Table S 3: Purified antibodies were used in this study   \| **Target** \|  \| **Catalog No.** \| **Company** \| \| --- \| --- \| --- \| --- \| \| F4/80 \| immunohistochemistry staining \| [MCA497](https://www.bio-rad-antibodies.com/mouse-f4-80-antibody-cl-a3-1-mca497g.html)RT \| Bio-Rad \| \| Gout anti-rat \| immunohistochemistry staining \| AP136P \| Sigma-Aldrich \| \| AMPK \| western blot \| Sc-74461 \| Santa Cruz \| \| p-AMPK \| western blot \| Sc-33524 \| Santa Cruz \| \| P38 \| western blot \| Sc-535 \| Santa Cruz \| \| p-P38 \| western blot \| Sc-166182 \| Santa Cruz \| \| JNK \| western blot \| Sc-7345 \| Santa Cruz \| \| p-JNK \| western blot \| Sc-6254 \| Santa Cruz \| \| Anti-mouse \| western blot \| Sc-2005 \| Santa Cruz \| \| Anti-rabbit \| western blot \| ab97051 \| abcam \| \| NF-κB p65 \| western blot \| ab16502 \| abcam \| \| TNF- alpha \| western blot \| Sc-130349 \| Santa Cruz \| \| β- Actin \| western blot \| Sc-47778 \| Santa Cruz \| \| Protein Ladder \| western blot \| 84785 \| Thermo Scientific \| | | | | |

**Supplementary Figure legends**

**Supplementary Figure S1: Food intake (g diet/ mouse/ day).** All data were analyzed by one-way ANOVA, Tukey post-test. Values are expressed as means ± SD. NS= no statistically significant difference between groups.

**Supplementary Figure S2: Cycle Threshold** (**CT) value of β-Actin mRNA.** All data were analyzed by one-way ANOVA, Tukey post-test. Values are expressed as means ± SD. NS= no statistically significant difference between groups.

**Supplementary Figure S3:** Full-length gels and western blots of (a) phospho-P38, (b) P38, (c) phospho-JNK, (d) JNK, (e) phospho-AMPK, (f) AMPK, (g) NF-κB (p65), (h) TNF-α (i) β-actin in skeletal muscle of NCD, HFD and HFD+RES groups.

**Supplementary Figure S1:**


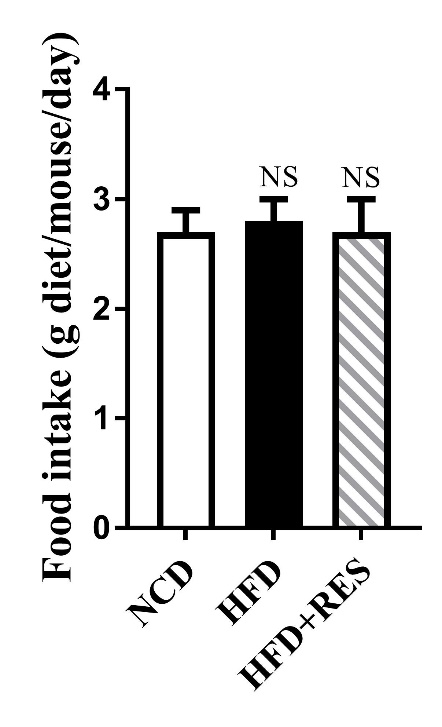


**Supplementary Figure S2:**


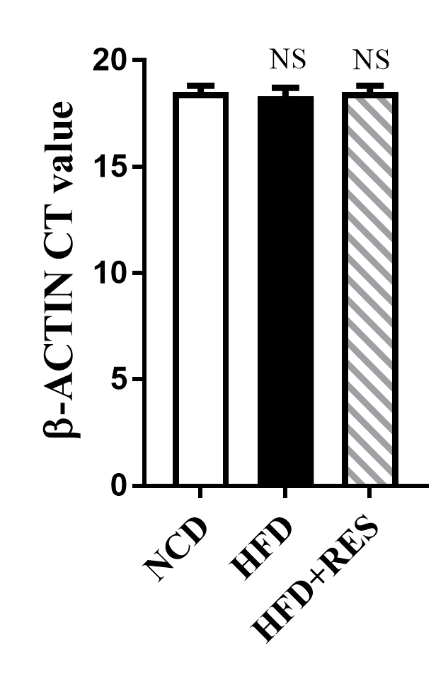


**Supplementary Figure S3:**


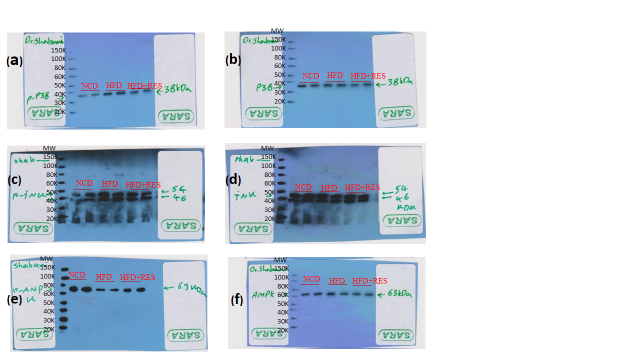

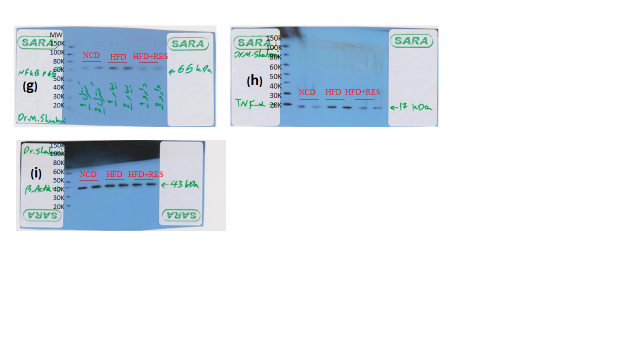

Supplement: Supplementary file 1 — Supplementary information. [file 41598_2020_60185_MOESM1_ESM.docx]
